# PPCRKB: a risk factor knowledge base of postoperative pulmonary complications

**DOI:** 10.1093/database/baae054

**Published:** 2024-07-19

**Authors:** Jianchao Duan, Peiyi Li, Aibin Shao, Xuechao Hao, Ruihao Zhou, Cheng Bi, Xingyun Liu, Weimin Li, Huadong Zhu, Guo Chen, Bairong Shen, Tao Zhu

**Affiliations:** Department of Anesthesiology, West China Hospital, Sichuan University, No. 37th, Guoxue Alley, Wuhou District, Chengdu, Sichuan 610041, China; Laboratory of Anesthesia and Critical Care Medicine, National-Local Joint Engineering Research, West China Hospital, Sichuan University, No. 37th, Guoxue Alley, Wuhou District, Chengdu, Sichuan 610041, China; Research Unit for Perioperative Stress Assessment and Clinical Decision, Chinese Academy of Medical Sciences (2018RU012), West China Hospital, Sichuan University, No. 37th, Guoxue Alley, Wuhou District, Chengdu, Sichuan 610041, China; Emergency Department, State Key Laboratory of Complex Severe and Rare Diseases, Peking Union Medical College Hospital, Chinese Academy of Medical Science and Peking Union Medical College, 1 Shuai Fu Yuan, Beijing 100730, China; Department of Anesthesiology, West China Hospital, Sichuan University, No. 37th, Guoxue Alley, Wuhou District, Chengdu, Sichuan 610041, China; Laboratory of Anesthesia and Critical Care Medicine, National-Local Joint Engineering Research, West China Hospital, Sichuan University, No. 37th, Guoxue Alley, Wuhou District, Chengdu, Sichuan 610041, China; Research Unit for Perioperative Stress Assessment and Clinical Decision, Chinese Academy of Medical Sciences (2018RU012), West China Hospital, Sichuan University, No. 37th, Guoxue Alley, Wuhou District, Chengdu, Sichuan 610041, China; Institutes for Systems Genetics, Frontiers Science Center for Disease-Related Molecular Network, West China Hospital, Sichuan University, Xinchuan Road 2222, Chengdu, Sichuan 610041, China; Department of Anesthesiology, West China Hospital, Sichuan University, No. 37th, Guoxue Alley, Wuhou District, Chengdu, Sichuan 610041, China; Laboratory of Anesthesia and Critical Care Medicine, National-Local Joint Engineering Research, West China Hospital, Sichuan University, No. 37th, Guoxue Alley, Wuhou District, Chengdu, Sichuan 610041, China; Department of Anesthesiology, West China Hospital, Sichuan University, No. 37th, Guoxue Alley, Wuhou District, Chengdu, Sichuan 610041, China; Laboratory of Anesthesia and Critical Care Medicine, National-Local Joint Engineering Research, West China Hospital, Sichuan University, No. 37th, Guoxue Alley, Wuhou District, Chengdu, Sichuan 610041, China; Institutes for Systems Genetics, Frontiers Science Center for Disease-Related Molecular Network, West China Hospital, Sichuan University, Xinchuan Road 2222, Chengdu, Sichuan 610041, China; Institutes for Systems Genetics, Frontiers Science Center for Disease-Related Molecular Network, West China Hospital, Sichuan University, Xinchuan Road 2222, Chengdu, Sichuan 610041, China; Department of Respiratory and Critical Care Medicine, West China Hospital, Sichuan University, No. 37th, Guoxue Alley, Wuhou District, Chengdu, Sichuan 610041, China; Institute of Respiratory Health, Frontiers Science Center for Disease-related Molecular Network, West China Hospital, Sichuan University, No. 37th, Guoxue Alley, Wuhou District, Chengdu, Sichuan 610041, China; Emergency Department, State Key Laboratory of Complex Severe and Rare Diseases, Peking Union Medical College Hospital, Chinese Academy of Medical Science and Peking Union Medical College, 1 Shuai Fu Yuan, Beijing 100730, China; Department of Anesthesiology, West China Hospital, Sichuan University, No. 37th, Guoxue Alley, Wuhou District, Chengdu, Sichuan 610041, China; Laboratory of Anesthesia and Critical Care Medicine, National-Local Joint Engineering Research, West China Hospital, Sichuan University, No. 37th, Guoxue Alley, Wuhou District, Chengdu, Sichuan 610041, China; Institutes for Systems Genetics, Frontiers Science Center for Disease-Related Molecular Network, West China Hospital, Sichuan University, Xinchuan Road 2222, Chengdu, Sichuan 610041, China; Department of Anesthesiology, West China Hospital, Sichuan University, No. 37th, Guoxue Alley, Wuhou District, Chengdu, Sichuan 610041, China; Laboratory of Anesthesia and Critical Care Medicine, National-Local Joint Engineering Research, West China Hospital, Sichuan University, No. 37th, Guoxue Alley, Wuhou District, Chengdu, Sichuan 610041, China

## Abstract

Postoperative pulmonary complications (PPCs) are highly heterogeneous disorders with diverse risk factors frequently occurring after surgical interventions, resulting in significant financial burdens, prolonged hospitalization and elevated mortality rates. Despite the existence of multiple studies on PPCs, a comprehensive knowledge base that can effectively integrate and visualize the diverse risk factors associated with PPCs is currently lacking. This study aims to develop an online knowledge platform on risk factors for PPCs (Postoperative Pulmonary Complications Risk Factor Knowledge Base, PPCRKB) that categorizes and presents the risk and protective factors associated with PPCs, as well as to facilitate the development of individualized prevention and management strategies for PPCs based on the needs of each investigator. The PPCRKB is a novel knowledge base that encompasses all investigated potential risk factors linked to PPCs, offering users a web-based platform to access these risk factors. The PPCRKB contains 2673 entries, 915 risk factors that have been categorized into 11 distinct groups. These categories include habit and behavior, surgical factors, anesthetic factors, auxiliary examination, environmental factors, clinical status, medicines and treatment, demographic characteristics, psychosocial factors, genetic factors and miscellaneous factors. The PPCRKB holds significant value for PPC research. The inclusion of both quantitative and qualitative data in the PPCRKB enhances the ability to uncover new insights and solutions related to PPCs. It could provide clinicians with a more comprehensive perspective on research related to PPCs in future.

**Database URL**: http://sysbio.org.cn/PPCs

## Introduction

Postoperative pulmonary complications (PPCs) represent a prevalent occurrence after anesthesia and surgical procedures (1–4). The prevalence of PPCs varies widely across the literature, with reported rates ranging from <1 to as high as 30% (2). The European Perioperative Clinical Outcome recommended definitions for PPCs, which encompass respiratory infection, respiratory failure, pleural effusion, atelectasis, pneumothorax, bronchospasm, aspiration pneumonitis, pneumonia, acute respiratory distress syndrome (ARDS) and pulmonary embolus (2, 5). There is a significant association between PPCs and increased mortality rates as well as prolonged hospital stays (6, 7). The development of PPCs also contributes to the escalation of healthcare costs, thereby imposing a substantial economic and healthcare resource burden (8).

In the current value-based care environment, it is critical to have methods to rapidly identify patients who are at the highest of PPCs and most likely to benefit from labor- or cost-intensive interventions. The development of PPCs is influenced by various factors, including those related to the patient, the surgical procedure and the administration of anesthesia (9–13). While certain factors, such as pre-existing pulmonary disease, are commonly recognized as risk factors for PPCs, others are inconsistently documented or continue to be a subject of debate, such as body mass index (BMI) (14–17). The prevention and management of PPCs are complicated by these factors. Therefore, an exhaustive compilation of risk factors may be useful for identifying potential etiology and preventing PPCs, as well as offering opportunities for further systematic analysis of the progression of PPC research.

Knowledge base systems can integrate a wide variety of information and knowledge, making it organized and intelligent (18, 19). A knowledge base provides various functions, including but not limited to searching, analytical tools, data visualization and links to other archival resources (20). It can effectively integrate quantitative and qualitative information, thereby facilitating the discovery of novel answers to queries (21). The knowledge base related to risk factors can aid in the collection of all studied risk factors and disease models, the study of interactions between factors, risk models and disease, and the development of personalized models for precision prediction (22, 23).

Despite the considerable amount of research that has been undertaken on PPCs, there is currently no comprehensive repository that integrates and presents all the risk factors associated with PPCs. Hence, we have developed a repository of knowledge on risk factors for PPCs, referred to as the Postoperative Pulmonary Complications Risk Factor Knowledge Base (PPCRKB). The knowledge base is available at http://sysbio.org.cn/PPCs. The primary objectives of this knowledge base are to categorize and present the risk and protective factors associated with PPCs, as well as to facilitate individualized prevention and management strategies for PPCs based on the specific requirements of each investigator.

## Methods

Prior to the commencement of data collection, consultation was sought from clinicians, experts in the field of life sciences and information experts. The purpose of these consultations was to ascertain the extent of data collection and to establish the categorization of risk factors. Throughout the data collection phase, we adjusted the enhancements to the methodology in accordance with the information presented in the article. Ultimately, the classification criteria were formulated and a data collection pipeline was constructed.

### Data collection

The compilation of disease categories for PPCs was derived from the definitions recommended by the European Perioperative Clinical Outcome. The method employed for collecting the risk factors associated with each type of PPCs involved querying the PubMed database using the disease name along with the keywords ‘risk’ and ‘predict’. The data collection period spanned from the inception of the database up until 1 March 2023. The complete search terms and search strategy can be found in Appendix 1.

**Figure 1.**
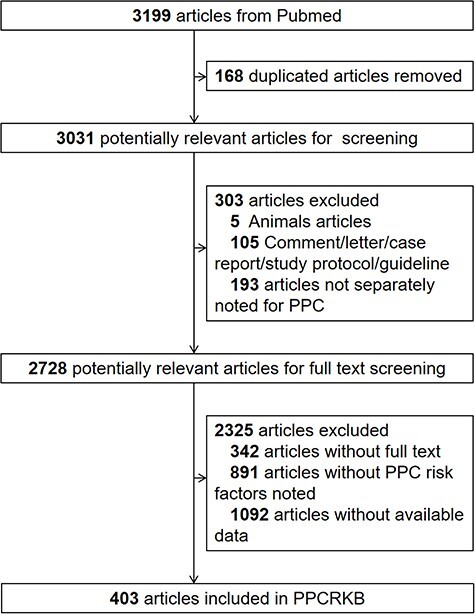
The flowchart of the data collection procession for PPCRKB.

Our inclusion criteria for studies in our database are as follows: (i) the study examined at least one form of PPCs and one risk factor; (ii) the conclusion of the study contains an unambiguous statement; (iii) any effect value, such as odds ratio, was provided. The following were included among the exclusion criteria: (i) the study used animals as subjects; (ii) the study combined PPCs with other diseases; (iii) the study of PPCs did not include any risk factors and (iv) the study did not report any effect magnitude with its 95% confidence intervals. Also excluded were comments, correspondence, case reports, study protocols, guidelines, and studies lacking entire texts. From January 1976 to January 2023, a total of 3031 studies were extracted from PubMed. After screening according to our inclusion and exclusion criteria, 403 articles were added to the PPCRKB knowledge base. Figure 1 illustrates the data collection process flowchart for the PPCRKB. The data were then manually stored in Excel (Microsoft Excel 2016), after which J.D. and P.L. double-checked the query results.

### Classification criteria

**Figure 2.**
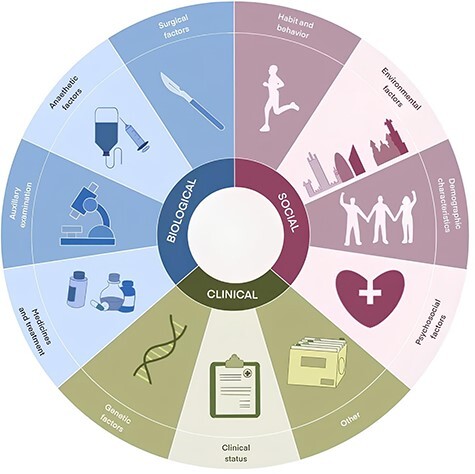
Eleven categories of PPCs’ risk factors.

We failed to discover that PPCs’ risk factors are presently categorized in a consistent approach based on our examination of the literature. In order to establish a coherent classification system, we engaged in discussions with clinicians and bioinformatics experts to develop criteria for categorizing risk factors associated with PPCs. This was necessary due to the potential variability in how these risk factors are described across different articles. The risk factors associated with PPCs were subsequently classified into 11 distinct categories: habit and behavior, surgical factors, anesthetic factors, auxiliary examination, environmental factors, clinical status, medicines and treatment, demographic characteristics, psychosocial factors, genetic factors, and others (Figure 2). Habit and behavior encompass various lifestyle factors, such as smoking, alcohol consumption, exercise and other related activities. Surgical factors encompass various elements, such as the specific type of surgery, duration of surgery and all associated procedural aspects. Anesthetic factors involve a wide range of anesthetic techniques and agents. Auxiliary examination contains blood routine examination, echocardiogram, chest X-ray and other clinical examinations. Environmental factors comprise residential environments and health care facilities, etc., while clinical status includes patients’ physiological and pathological conditions. Medicines and treatment involve treatment approaches and medication both during and after hospitalization. Demographic characteristics contain age, gender, BMI, etc. Psychosocial factors include social support, living conditions, psychosocial stressors, etc. Genetic factors primarily include genes and their variants. The factors that have not been enumerated previously are categorized as other factors.

### Data model

In order to establish a comprehensive knowledge base, we have constructed four tables utilizing the data that has been gathered. These tables are categorized as follows: factor Information (Table 1), PPC Information (Table 2), reference (Table 3) and statistical analysis (Table 4).

**Table 1.** Factor information

Attributes	Description	Examples
**Factor name**	Risk factor name	Age
**Description**	Description of factor and additional condition	>80 years
**F_ID**	Risk factor ID	2
**Factor type**	Risk factor type	Demographic characteristics
**Patient**	Study population	Patients who underwent scheduled or emergency surgery with general, neuraxial, or regional anesthesia.
**Surgery**	Procedure subtype and surgical approach	Scheduled or emergency surgery
**Association** ^a^	Whether this factor is a risk factor	Positive

F_ID, Factor Identity document;

a Association is based on the effect size in each reference.

**Table 2.** PPC information

Attributes	Description	Examples
**PPC definition**	The definition of PPC in the literature	Postoperative pulmonary complication within the first 7 postoperative days through review of chart, laboratory, and radiology data. These included: (1) respiratory failure requiring mechanical ventilation, (2) pneumonia, (3) atelectasis requiring bronchoscopic intervention, and (4) pneumothorax or pleural effusion requiring percutaneous intervention.
**Incidence**	The recorded incidence of PPC	27.1%
**PPC type**	Subtype of PPC	PPCs

**Table 3.** Information on reference

Attributes	Description	Examples
**PMID**	PubMed Unique Identifier	15 563 632
**Title**	The title of literature	Incidence of and risk factors for pulmonary complications after nonthoracic surgery
**Reference type**	The type of literature	Prospective study
**Publish year**	Year of publication	2005
**Study period**	The time horizon of the study	(2001,2003)
**Study area**	Country, areas or regions under study	Canada
**Sample**	Sample size	1055
**Conclusion comment**	Conclusions related to risk factors	Multivariate analyses revealed that four were independently associated with increased risk of pulmonary complications: age (odds ratio [OR] 5.9 for age >/= 65 years, p < 0.001), positive cough test (OR 3.8, P = 0.01), perioperative nasogastric tube (OR 7.7, p < 0.001), and duration of anesthesia (OR 3.3 for operations lasting at least 2.5 hours, p = 0.008). Thus, several perioperative factors predict an increased risk for pulmonary complications after elective nonthoracic surgery.

**Table 4.** Statistical analysis of each article

Attributes	Description	Examples
**Statistical method**	Data analysis approach and model	Multivariable logistic regression analysis
**Cut-off point**	Cut-off point	
**Sensitivity**	Sensitivity	
**Specificity**	Specificity	
**Positive predictive value**	Positive predictive value	
**Negative predictive value**	Negative predictive value	
**Ratio**	Ratio	
**Relative risk**	Relative risk	
**Hazard ratio**	Hazard ratio	
**Odds ratio**	Odds ratio	5.73
**AUC**	Area Under Curve	
**Other effect size**	aOR,, aHR, IRR, etc.	
**Confidence interval**	Confidence interval	(2.49,13.15)
***P*-value**	*P*-value	<0.001

### Knowledge base construction

**Figure 3.**
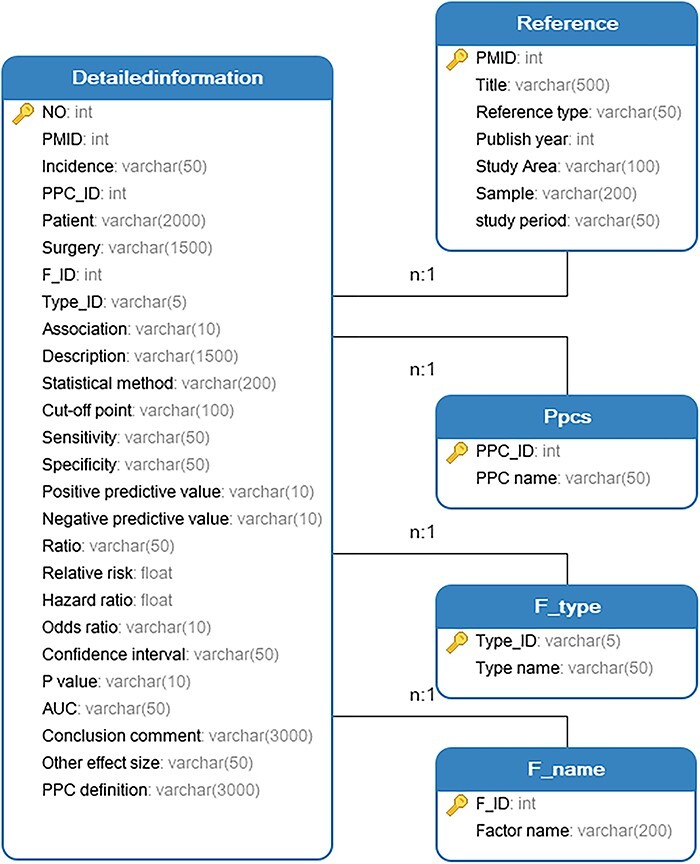
Unified modeling language class diagrams of PPCRKB.

The knowledge base was developed by considering various application scenarios, encompassing its functionality, interface and content. In order to establish our knowledge base, we employed the WAMP (Windows + Apache + MySQL + PHP) development environment. The entity relationships of the PPCRKB knowledge base are depicted in Figure 3 using unified modeling language class diagrams.

## Results

### Data statistics

The current iteration of PPCRKB contains nine distinct types of PPC: respiratory failure (125 risk factors, total of 244 records), pleural effusion (30 risk factors, total of 35 records), atelectasis (15 risk factors, total of 17 records), pneumothorax (15 risk factors, total of 17 records), pneumonia (239 risk factors, total of 560 records), ARDS (56 risk factors, total of 95 records), pulmonary edema(one risk factor, one record), pulmonary embolism (38 risk factors, total of 44 records) and PPCs (590 risk factors, total of 1660 records).

The PPCRKB encompasses a total of 915 risk factors. These risk factors are categorized into the following 11 categories based on characteristics: 9 habit and behavior, total of 99 records; 190 surgical factors, total of 652 records; 71 anesthetic factors, total of 203 records; 168 auxiliary examination, total of 400 records; 16 environmental factors, total of 45 records; 311 clinical status, total of 699 records; 69 medicines and treatment, total of 88 records; 20 demographic characteristics, total of 338 records; 3 psychosocial factors, total of 3 records; 6 genetic factors, total of 6 records; and 88 other factors, total of 140 records.

### Database schema

The PPCRKB consists of six pages, each serving a specific purpose. The homepage provides an introduction to the knowledge base. The search page allows users to conduct basic and advanced searches. The statistics page presents data visualization to enhance understanding. The update page enables users to submit new risk factors. The help page offers guidelines for search and update processes. Lastly, the ‘About Us’ page provides information about the team involved and the address of the knowledge base.

### Web interface and function

#### Home page

**Figure 4.**
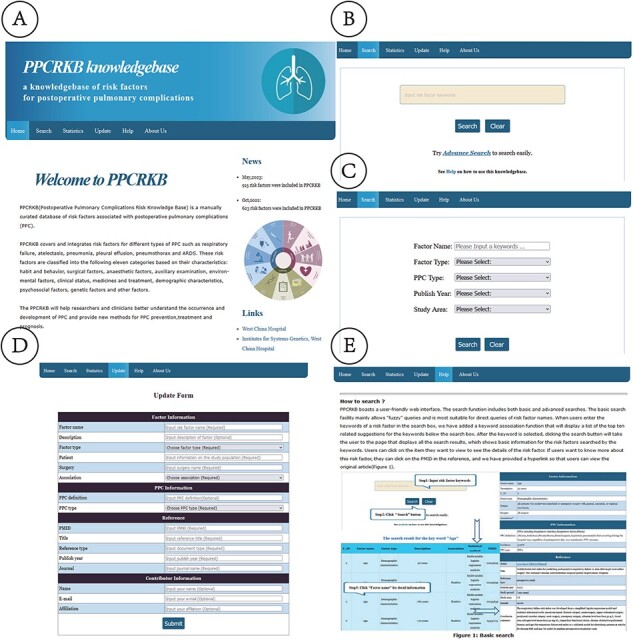
Web interface of PPCRKB. (A) Home page; (B) Basic search page; (C) Advanced search page; (D) Update page; (E) Help page.

The contents and functions of the database platform are introduced on the home page, as depicted in Figure 4A. This aims to assist users in effectively utilizing PPCRKB. This page also demonstrates our efforts to continuously update the PPCRKB, thereby ensuring that this knowledge base remains current with the latest information.

#### Search page

The PPCRKB platform features a web interface that is designed to be easily navigable and accessible for users. The search functionality encompasses both rudimentary and sophisticated search options. The primary search function primarily accommodates imprecise queries and is best suited for direct inquiries of risk factor names (Figure 4B). We have implemented a keyword association feature that generates a list of the 10 most relevant keywords when users input risk factor keywords into the search box. These associated keywords are then displayed below the search box. Once the user has selected the keyword, they can proceed by clicking the search button, which will redirect them to a page that presents all the search results. This page will provide users with basic information on the searched risk factors. Additionally, it will automatically generate statistical analysis charts related to these factors, enabling users to swiftly identify associated studies and make preliminary assessments regarding their potential as risk factors. To access detailed information regarding a specific risk factor, users can click on the respective item of interest. If users desire to acquire further information regarding this risk factor, they may access the PMID in the reference. Additionally, we have included a hyperlink for users to conveniently access the original article. The advanced search feature enables users to perform combined searches. Users can enhance the precision of their search results by combining and specifying various search terms, as depicted in Figure 4C.

#### Statistics page

**Figure 5.**
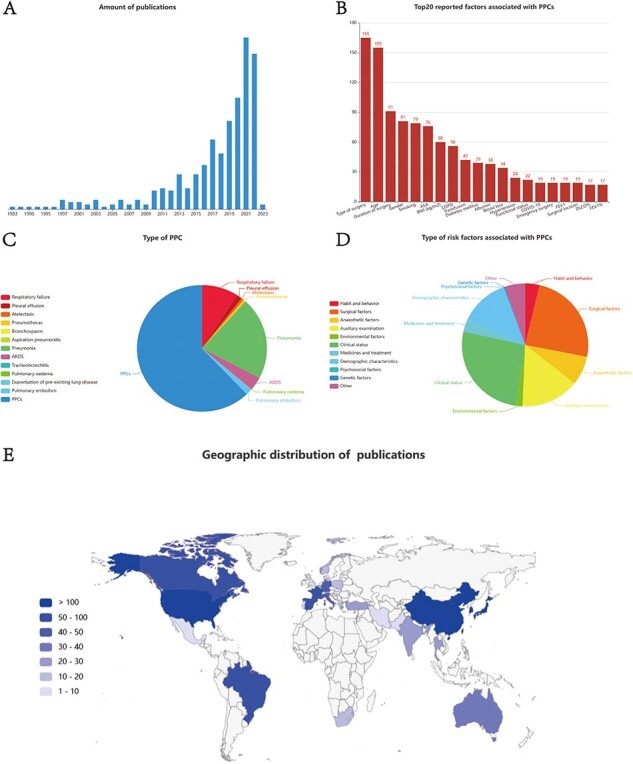
Statistics page of PPCRKB. (A) Chart shows the number of studies by year of publications; (B) Chart shows Top20 reported factors associated with PPCs; (C) Chart shows the type of PPC included in PPCRKB; (D) Chart shows the type of risk factors included in PPCRKB; (E) Chart shows the geographic distribution of publications.

A statistical analysis was conducted on the data within the PPCRKB, and the resulting findings were presented on the statistics page. Based on an analysis of the distribution patterns of publication years, it is evident that there were relatively fewer studies focusing on the risk factors associated with PPCs in previous decades. However, it is noteworthy that the number of relevant articles has experienced a significant increase in recent years (Figure 5A). We also identified the top 20 reported risk factors related to PPCs in accordance with the number of risk variables whose names were included in the research (Figure 5B), which may help to highlight the research objectives and hotspots in the investigation of PPCs’ risk factors. PPCs make up the majority of the 11 different forms of PPC in the existing PPCRKB, accounting for ∼62.1%, followed by pneumonia (20.95%) (Figure 5C). Because no more relevant information could be reviewed, the risk factors for bronchospasm, aspiration pneumonitis, tracheobronchitis, and worsening of pre-existing lung illness were not gathered in this knowledge base. The examination of the various risk variables associated with PPCs was conducted in Figure 5D, with particular emphasis on the clinical state and surgical factors as the primary contributors. The spatial arrangement of the study’s data is depicted in Figure 5E. The primary focus of the investigations was centered in the USA, followed by subsequent research conducted in China, Japan, Korea and France.

#### Update page

The update page (Figure 4D) offers users the opportunity to add new risk factors to the table. The updated form consists of various items, including risk factor name, description, factor type, patient, surgery, association, PPC definition, PPC type, PMID, article title, reference type, publish year, journal, contributor’s name, contributor’s e-mail and contributor’s affiliation. Every item is accompanied by a detailed description and an indication of whether it is a compulsory choice. Upon the completion of the user’s submission of the form, the data will be promptly transferred and stored in our database. The inclusion of new factors into the PPCRKB will occur after a thorough manual examination and meticulous evaluation. In addition to user-contributed data, we will conduct a comprehensive update of the knowledge repository annually. This systematic process will ensure the continuous enhancement and reliability of our database.

#### Help page

On the help page, the utilization of the knowledge base is introduced (Figure 4E). This module facilitates users in efficiently acquiring comprehension of our knowledge base and in submitting novel factors for enhancement.

#### About us page

Figure 4F provides an overview of the Institutes for Systems Genetics and Anesthesia & Operation Center at West China Hospital, Sichuan University.

## Discussion

The PPCRKB is the first knowledge base encompassing risk factors associated with PPCs, offering users comprehensive access to information of these risk factors. The data were gathered and validated by proficient experts to ensure precision. PPCRKB differs from other perioperative databases, including the National Cancer Database (NCDB) and the Surgical Quality Improvement Program (NSQIP) in many key ways. PPCRKB’s main objective is to systematically collect and publish information on the risk factors for PPCs. Instead of acting as a comprehensive database of perioperative information, its emphasis is on providing insights and advice for the prevention and treatment of PPCs (24, 25). Instead of using clinical data, PPCRKB uses summary-level information that may be accessed in scholarly publications. The PPCRKB has data visualization tools that are exclusive to this database and provides comprehensive information on risk variables.

Compared with prior research conducted in this field, the PPCRKB exhibits several distinctions and notable advantages. Initially, a methodical gathering of current evidence pertaining to the risk factors associated with PPCs was undertaken. In contrast to a multitude of machine-based databases, manually collected data exhibits greater precision and comprehensiveness (22). The PPCRKB will undergo annual updates to synchronize with the publication cycle of relevant literature and the update frequency of other databases, thereby ensuring its continual currency and reliability. Furthermore, the PPCRKB encompasses a wider range of risk factors, including both non-risk factors and risk models. Furthermore, the study also differentiated between risk factors, protective factors, and factors that were not found to have a statistically significant association with PPCs. In addition, PPCRKB offers enhanced search functionalities, such as fuzzy search and list search, thereby enhancing its overall usability. Moreover, the PPCRKB platform offers users the capability to effectively visualize and analyze static data through a user-friendly web interface. Lastly, the knowledge base exhibits extensibility, allowing for future updates and sharing.

The preliminary analysis of risk factors in the PPCRKB has identified a discrepancy in the existing literature regarding the classification of certain factors as risk factors. For instance, variables such as middle age, history of chronic obstructive pulmonary disease, as well as obesity have been subject to conflicting interpretations in terms of their association with PPCs risk. Take obesity as an example, Wightman *et al*. (26) observed that individuals who are underweight face a higher likelihood of experiencing PPCs and other complications. Conversely, the risk associated with undergoing surgery for patients who are overweight or obese is comparable to that of individuals with a normal BMI. Another study revealed a positive correlation between obesity, specifically morbid obesity, and the likelihood of experiencing pulmonary complications (27). The researchers observed that as obesity levels escalated, the risk of encountering medical complications, particularly those related to the respiratory system, also increased. Li *et al*. (28) conducted a study that showcased the favorable effects of higher BMI and obesity on the in-hospital outcomes and long-term survival of patients who underwent lung cancer surgery. This phenomenon, commonly referred to as the ‘obesity paradox’, has the potential to manifest in the context of lung cancer surgery. Further investigation is necessary to elucidate the underlying reasons for these conflicting results. Users could retrieve comprehensive literature and detailed information pertaining to the topic of ‘obesity’ by accessing the PPCRKB database. From this perspective, the data obtained from the PPCRKB can serve as a crucial empirical evidence for future academic investigations into PPCs.

The utilization of PPCRKB is anticipated to play a significant role in clinical research and the development of personalized treatment strategies for patients with PPCs. The PPCRKB is a meticulously curated and an all-encompassing repository dedicated to providing comprehensive information on PPCs, which enables clinicians to utilize specific factors in order to facilitate the process of risk identification and formulating prevention plans. Furthermore, it is crucial to note that PPCRKB has the potential to offer valuable insights for constructing the PPCs ontology. And it is of great importance to perform data normalization, dismantle data silos, and facilitate data sharing to foster research advancements in the field of PPCs. Additionally, the organized data within the PPCRKB will be transformed into a knowledge atlas, offering a novel method for analyzing risk factors and preventing PPCs (Appendix 2). While the current iteration of our knowledge atlas does not yet encompass these functionalities, planned enhancements aim to realize this vision. These enhancements involve integrating treatment strategies and their effectiveness in mitigating PPCs, accommodating longitudinal data for visualizing changes in risk factor profiles over time, and incorporating machine learning models to identify crucial points and trends in the data. In the future, the atlas will enable precise risk assessment and stratification, integrating predictive models to tailor interventions and reduce PPCs occurrences. It will facilitate evidence-based decision-making, providing clinicians with insights into the effectiveness of different treatment options for personalized care. Moreover, the atlas will highlight areas lacking in-depth investigation, guiding future research towards filling these knowledge gaps. This will not only contribute to a deeper comprehension of PPCs but also to the development of more effective prevention and management strategies. This evolution from our present capabilities to future applications underscores our commitment to bridging the gap between academic research and clinical practice.

To date, our data collection has been limited to risk factors sourced exclusively from PubMed, without incorporating information from other databases, such as EMBASE, Web of Science, and others. Hence, it is possible certain data that satisfy our inclusion criteria may not be incorporated into the PPCRKB, thereby resulting in the incompleteness of the knowledge base. Besides, in our study, we employed manual extraction for data retrieval, prioritizing accuracy but at the cost of time and significant human effort. We also explored using ChatGPT for extraction, aiming to enhance efficiency. While ChatGPT excelled in quickly identifying basic information, its performance on complex data was less reliable, showing inconsistencies and inaccuracies. This led to a need for frequent manual corrections, reducing the anticipated efficiency gains. Given these limitations, our experience underscores the necessity for developing a new model that combines the precision of manual methods with the speed of automated tools like ChatGPT, to optimize the knowledge extraction process for future research endeavors. Furthermore, our knowledge base has limited inclusion of genetic information related to postoperative pulmonary complications. This is mainly due to the scarcity of genetic studies specifically focusing on postoperative pulmonary complications. The lack of research in this area hampers our ability to comprehensively understand the genetic risk factors associated with these complications. Future endeavors should strive to conduct more extensive genomic research to supplement and enhance our knowledge base. In the forthcoming iteration of PPCRKB, our focus will be on enhancing the comprehensiveness and accuracy of the data accumulated within the database. In addition, the reliability of a risk factor, if it is only reported by a limited number of publications, should be noted and interpreted with caution. To effectively tackle this issue, further research aims to establish a multi-criteria evaluation system to ensure the reliability of the risk factors included in PPCRKB by considering the journal’s impact, sample size, etc. The authors of this study aim to consistently enhance the PPCRKB platform, optimize database functionality, and develop individualized prediction models for precise PPCs (29).

## Conclusion

The integration of the ‘Internet +’ and ‘AI’ (artificial intelligence) has the potential to facilitate the digitalization of traditional medicine, a crucial aspect in the realm of medicine and healthcare. The advent of a medical knowledge base heralds a transformative era in the realm of disease prevention and therapeutic interventions. A knowledge base, known as the PPCRKB, has been developed to facilitate the risk identification, accurate prevention and treatment of PPCs in the future. The PPCRKB holds significant value for PPCs research. Our ongoing efforts involve continuously updating the knowledge base, integrating visual statistical functions, and constructing personalized models to forecast the likelihood of PPCs. These endeavors have the potential to provide clinicians with a more comprehensive perspective on research related to PPCs.

## Supplementary Material

baae054_Supp

## Data Availability

All data relevant to this study are incorporated into the article or available online in the PPCRKB (http://sysbio.org.cn/PPCs).

## Appendix 1 Search terms and search strategy

1: ((Postoperative complications [Title/Abstract]) AND (risk [Title/Abstract])) AND (pulmonary[Title/Abstract])

2: (Postoperative pulmonary complications[Title/Abstract]) AND (predict[Title/Abstract])

3: (PPCs[Title/Abstract]) AND (risk[Title/Abstract])

4:(Postoperative respiratory infection[Title/Abstract]) AND (risk [Title/Abstract])

5:(Postoperative respiratory failure[Title/Abstract]) AND (risk [Title/Abstract])

6:(Postoperative respiratory failure[Title/Abstract]) AND (predict [Title/Abstract])

7:(postoperative pleural effusion[Title/Abstract]) AND (risk[Title/Abstract])

8:(postoperative atelectasis[Title/Abstract]) AND (risk[Title/Abstract])

9:(postoperative atelectasis[Title/Abstract]) AND (predict[Title/Abstract])

10:(postoperative pneumothorax[Title/Abstract]) AND (predict[Title/Abstract])

11:(postoperative pneumothorax[Title/Abstract]) AND (risk[Title/Abstract])

12:(postoperative bronchospasm[Title/Abstract]) AND (risk[Title/Abstract])

13:(postoperative bronchospasm[Title/Abstract]) AND (predict[Title/Abstract])

14:(postoperative pneumonia[Title/Abstract]) AND (risk[Title/Abstract])

15:(postoperative pneumonia[Title/Abstract]) AND (predict[Title/Abstract])

16:(postoperative ARDS[Title/Abstract]) AND (predict[Title/Abstract])

17:(postoperative ARDS[Title/Abstract]) AND (risk[Title/Abstract])

18:(postoperative tracheobronchitis[Title/Abstract]) AND (risk[Title/Abstract])

19:(postoperative tracheobronchitis[Title/Abstract]) AND (predict[Title/Abstract])

20:(postoperative pulmonary oedema[Title/Abstract]) AND (risk[Title/Abstract])

21:(postoperative pulmonary embolism[Title/Abstract]) AND (risk[Title/Abstract])[Title/Abstract]) AND (risk[Title/Abstract])

22:(postoperative pulmonary embolism[Title/Abstract]) AND (predict[Title/Abstract])[Title/Abstract]) AND (risk[Title/Abstract])

## Appendix 2

Knowledge Atlas of PPCs and related factors. The graph illustrates the associations between postoperative pulmonary complications (PPCs) and related factors within a weighted network diagram. Nodes represent PPCs and factors, marked as PPC IDs and F IDs, with their size indicating connection frequency, highlighting the prevalence and importance of each element. Edges, color-coded to differentiate relationship types—orange for risk factors, green for protective, and grey for neutral—map the network. PPC nodes in light coral and factor nodes in light blue visually distinguish the components, offering insight into the network of PPCs and their influencers.
