# Supplementary figures and images for: PPCRKB: a risk factor knowledge base of postoperative pulmonary complications
Source: Database (Oxford). 2024 Jul 19;2024:baae054. doi: 10.1093/database/baae054 (PMC11259045; doi:10.1093/database/baae054)

**Figure R1** Comparison of ChatGPT and manually extraction of literature data.

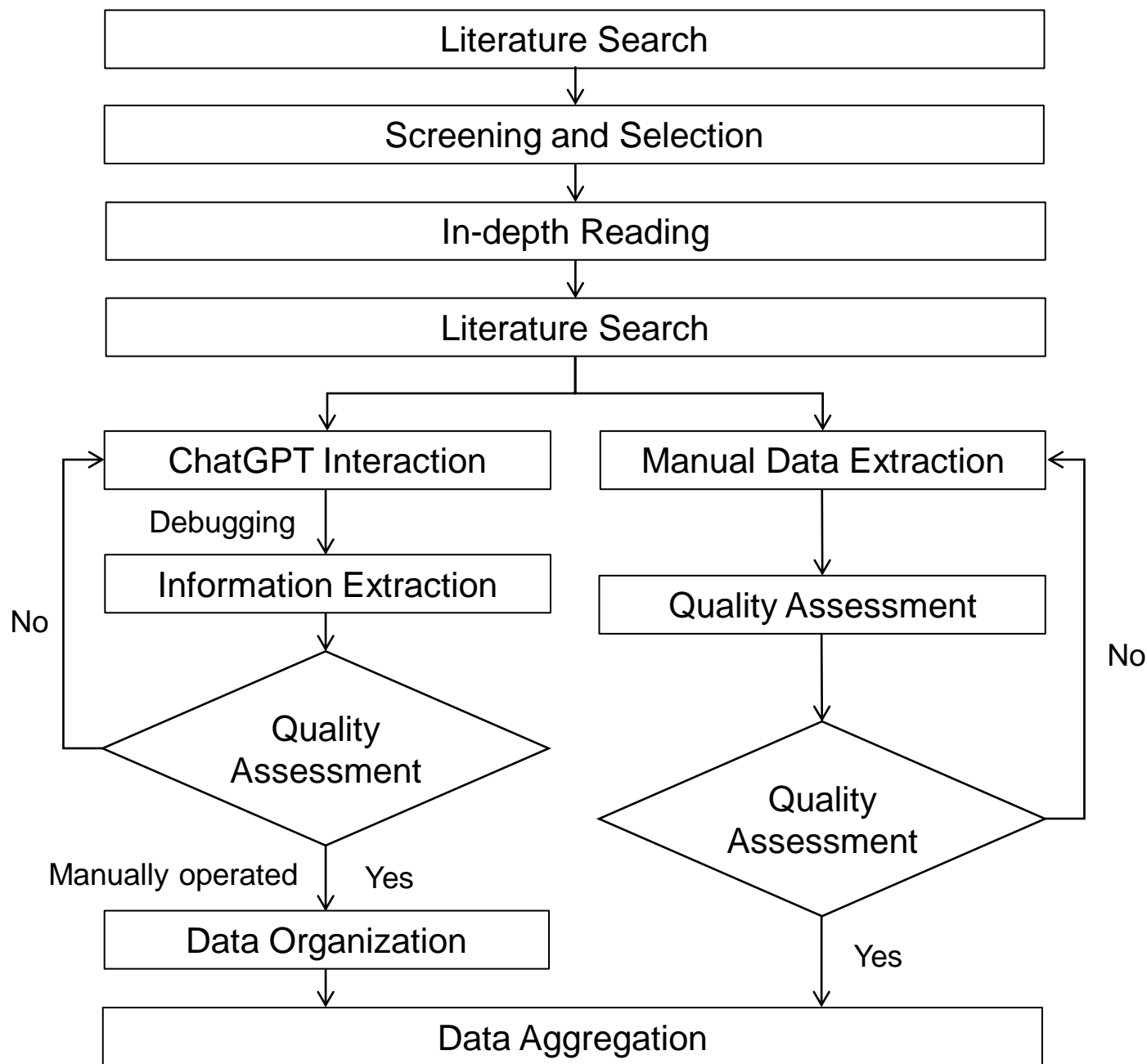

Supplement: baae054_Supp [file baae054_supp.zip › suppl_data/Fig. R1 Comparison of ChatGPT and manually extraction of literature data..pdf]

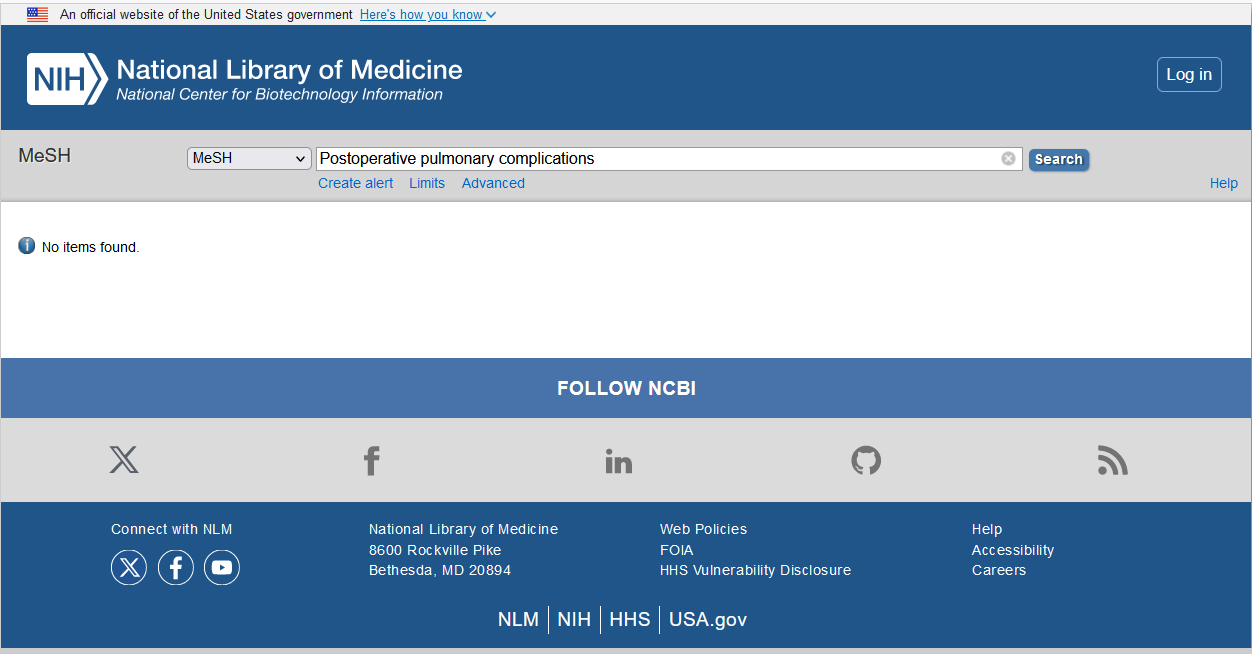

Supplement: baae054_Supp [file baae054_supp.zip › suppl_data/Fig. R3 The retrieval results of PPCs searched in the MeSH..png]

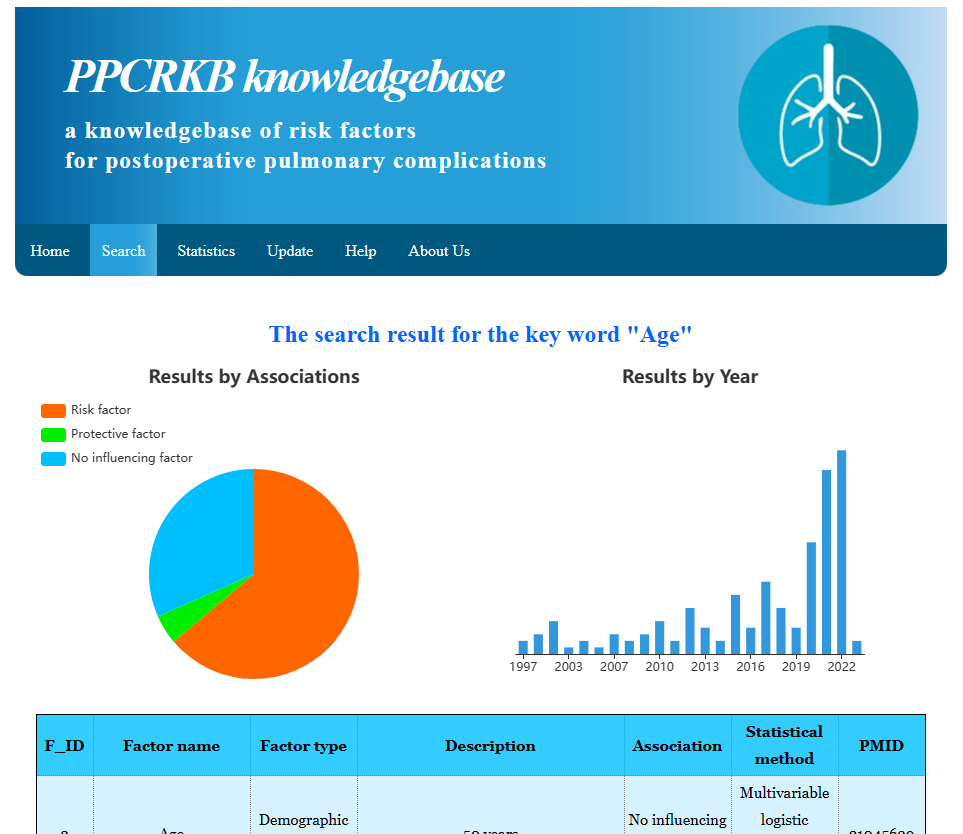

Supplement: baae054_Supp [file baae054_supp.zip › suppl_data/Fig. R4 Dynamic visualization of specific risk factors associated with PPCs..png]

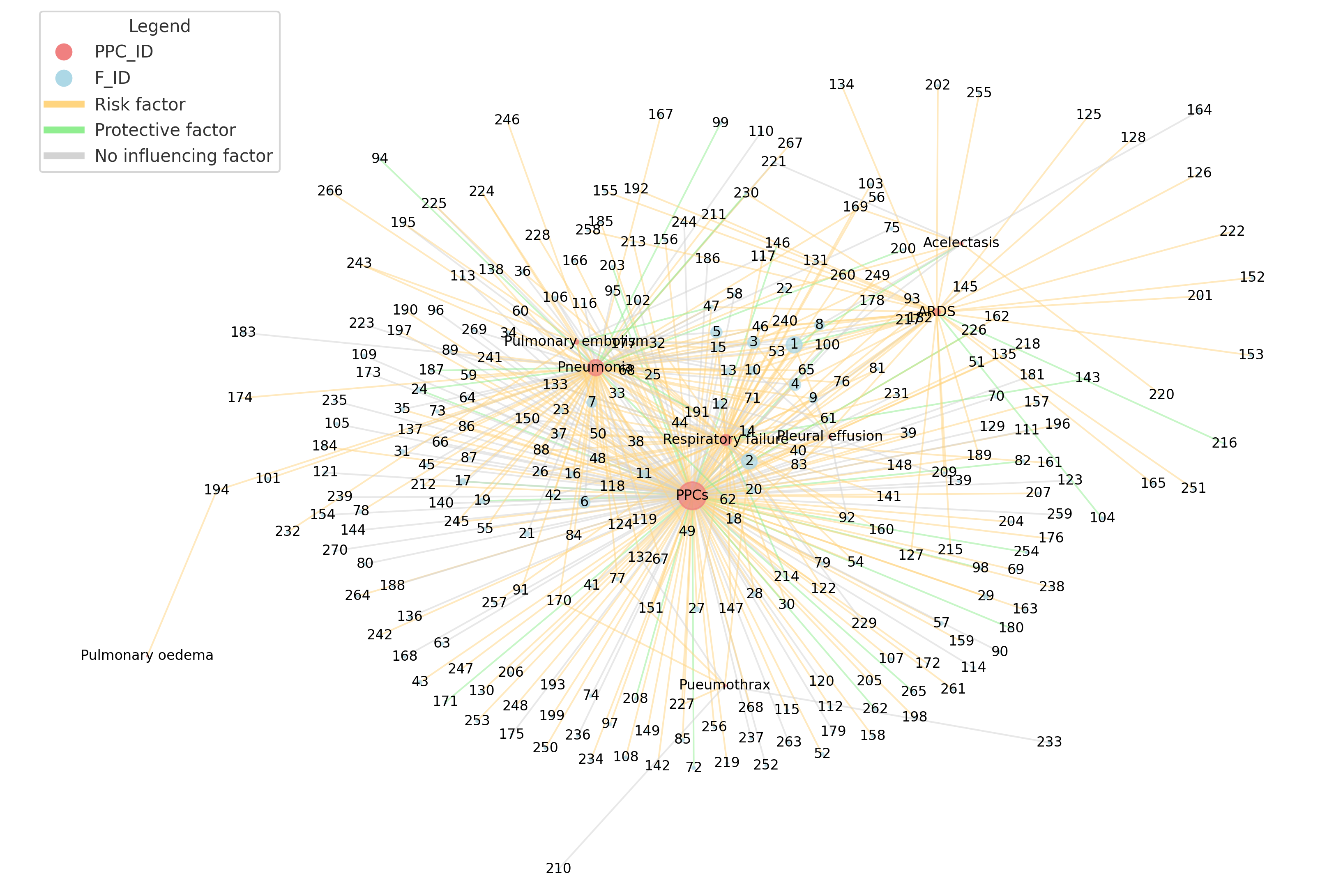

Supplement: baae054_Supp [file baae054_supp.zip › suppl_data/Fig. S1 The graph illustrates the associations between postoperative pulmonary complications (PPCs) and related factors within a weighted network diagram. Nodes represent PPCs and factors, marked as PPC ID.png]
