# Supplementary material for: PPCRKB: a risk factor knowledge base of postoperative pulmonary complications
Source: Database (Oxford). 2024 Jul 19;2024:baae054. doi: 10.1093/database/baae054 (PMC11259045; doi:10.1093/database/baae054)
Supplement: baae054_Supp [file baae054_supp.zip › suppl_data/Appendix 1.doc]

**PPCRKB: A risk factor knowledge base of postoperative pulmonary complications**

**An original study**

**Appendix 1 Search terms and search strategy**

1: ((Postoperative complications[Title/Abstract]) AND (risk [Title/Abstract])) AND (pulmonary[Title/Abstract])

2: (Postoperative pulmonary complications[Title/Abstract]) AND (predict[Title/Abstract])

3: (PPCs[Title/Abstract]) AND (risk[Title/Abstract])

4:(Postoperative respiratory infection[Title/Abstract]) AND (risk [Title/Abstract])

5:(Postoperative respiratory failure[Title/Abstract]) AND (risk [Title/Abstract])

6:(Postoperative respiratory failure[Title/Abstract]) AND (predict [Title/Abstract])

7:(postoperative pleural effusion[Title/Abstract]) AND (risk[Title/Abstract])

8:(postoperative atelectasis[Title/Abstract]) AND (risk[Title/Abstract])

9:(postoperative atelectasis[Title/Abstract]) AND (predict[Title/Abstract])

10:(postoperative pneumothorax[Title/Abstract]) AND (predict[Title/Abstract])

11:(postoperative pneumothorax[Title/Abstract]) AND (risk[Title/Abstract])

12:(postoperative bronchospasm[Title/Abstract]) AND (risk[Title/Abstract])

13:(postoperative bronchospasm[Title/Abstract]) AND (predict[Title/Abstract])

14:(postoperative pneumonia[Title/Abstract]) AND (risk[Title/Abstract])

15:(postoperative pneumonia[Title/Abstract]) AND (predict[Title/Abstract])

16:(postoperative ARDS[Title/Abstract]) AND (predict[Title/Abstract])

17:(postoperative ARDS[Title/Abstract]) AND (risk[Title/Abstract])

18:(postoperative tracheobronchitis[Title/Abstract]) AND (risk[Title/Abstract])

19:(postoperative tracheobronchitis[Title/Abstract]) AND (predict[Title/Abstract])

20:(postoperative pulmonary oedema[Title/Abstract]) AND (risk[Title/Abstract])

21:(postoperative pulmonary embolism[Title/Abstract]) AND (risk[Title/Abstract])[Title/Abstract]) AND (risk[Title/Abstract])

22:(postoperative pulmonary embolism[Title/Abstract]) AND (predict[Title/Abstract])[Title/Abstract]) AND (risk[Title/Abstract])
